# A Novel Cooperative Localization Method Based on IMU and UWB

**DOI:** 10.3390/s20020467

**Published:** 2020-01-14

**Authors:** Yongqiang Han, Chenchen Wei, Rong Li, Jingzhe Wang, Huan Yu

**Affiliations:** 1 School of Automation, Beijing Institute of Technology, Beijing 100081, China; Wei15620608924@163.com (C.W.); 2120170954@bit.edu.cn (J.W.); yuhuan.bit@gmail.com (H.Y.); 2 The 95894 Unit, PLA, Beijing 102211, China; livvyrong@163.com

**Keywords:** cooperative localization, dead reckoning, inertial measurement, ultra-wideband, pose estimation

## Abstract

In this paper, a range-based cooperative localization method is proposed for multiple platforms of various structures. The localization system of an independent platform might degrade or fail due to various reasons such as GPS signal-loss, inertial measurement unit (IMU) accumulative errors, or emergency reboot. It is a promising approach to solve this problem by using information from neighboring platforms, thus forming a cooperative localization network that can improve the navigational robustness of each platform. Typical ranging-based ultra-wideband (UWB) cooperative localization systems require at least three auxiliary nodes to estimate the pose of the target node, which is often hard to meet especially in outdoor environment. In this work, we propose a novel IMU/UWB-based cooperative localization solution, which requires a minimum number of auxiliary nodes that is down to 1. An Adaptive Ant Colony Optimization Particle Filter (AACOPF) algorithm is customized to integrate the dead reckoning (DR) system and auxiliary nodes information with no prior information required, resulting in accurate pose estimation, while to our knowledge the azimuth have not been estimated in cooperative localization for the insufficient observation of the system. We have given the condition when azimuth and localization are solvable by analysis and by experiment. The feasibility of the proposed approach is evaluated through two filed experiments: car-to-trolley and car-to-pedestrian cooperative localization. The comparison results also demonstrate that ACOPF-based integration is better than other filter-based methods such as Extended Kalman Filter (EKF) and traditional Particle Filter (PF).

## 1. Introduction

**Figure 1:**
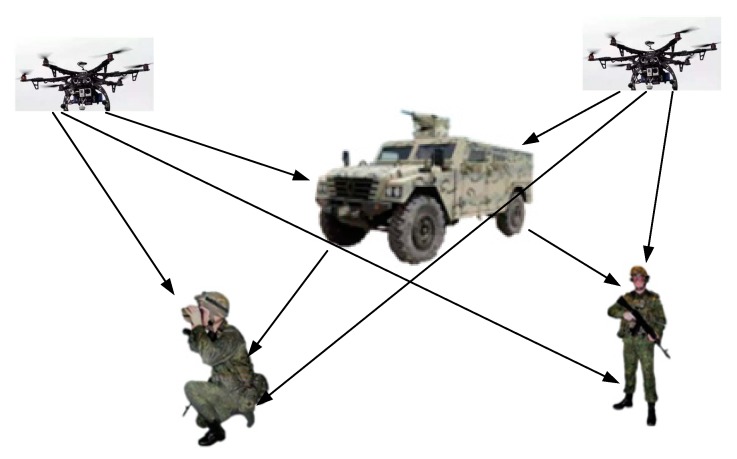
The scenario application model of collaborative localization.

The cooperative operation among manned and unmanned platforms is becoming increasingly demanding with the development of navigation, communication, and intelligent control technologies, etc. Most robotic platforms or even human are equipped with navigation devices such as Global Navigation Satellite System (GNSS) and inertial measurement unit (IMU) [1]. However, positioning tasks become difficult in the face of IMU error accumulation, signal occlusion, and environmental interference (smoke, buildings, forests, canyons, etc.). The concept of joint localization begins to gain in popularity [2,3]. Unlike positioning platforms with a single node, cooperative localization fully exploits positioning information exchanged in a multi-node ad hoc network. Not only can it lead to more accurate estimates for the position of the target node, but also expand the coverage and enhance the overall stability of the network [4]. Figure 1 illustrates an application scenario in which soldiers and unmanned vehicles are accurately localized under a collaborative network.

Current mainstream schemes of collaborative localization fall into two categories in generally, one of which employs Bluetooth [5], Wi-Fi [6], ZigBee [7], ultrasonic [8], infrared [9], UWB [10] based on angle of arrival (AOA), time of arrival (TOA), and time difference (TDOA) methods under such models of geometry as trilateration, triangulation, and hyperbolas, which entails at least 3 auxiliary nodes together with range information. Unfortunately, this requirement cannot be met in many cases, and one shortcoming of these schemes is that the localization of the target node is completed only according to the information of the auxiliary node, while the information about the target node is ignored. Reference [11] can attain centimeter-level positioning accuracy using multiple UWB transceivers to co-locate but with a considerable number of nodes involved, thus incurring high costs. Reference [12] uses geometric features of translation and rotation to estimate the positioning error in inertial navigation of aircraft. Usually, these commonly employed nodes location methods entail additional auxiliary nodes, causing waste of resources and introducing extra costs, computational load, and communication load of the entire network.

Another type of formulation, rather than relying merely on auxiliary nodes based on geometry, is based on fusion of inertial readings from an IMU deployed on the target node and ranging information from auxiliary nodes. IMU is a self-contained system that measures linear and angular motion usually with gyroscopes and accelerometers independent of external assistance, it is immune to deception and signal jamming [13]. Therefore, IMU is capable of performing autonomous localization, but integration of inertial measurements is bound to drift. Typical combinations include Wi-Fi/IMUs, cameras/IMUs, UWB/IMUs, etc. In this paper, we focus on IMU/UWB collaborative localization in view of the properties of the UWB signal including low power consumption, immunity to channel fading (such as multipath, non-line-of-sight (NLOS), etc.), powerful signal penetration, and high precision.

Numerous studies of research have been done on UWB/IMU collaborative localization. Most UWB cooperative localization systems assume prior knowledge on the positions of no less than two auxiliary nodes. Reference [14] proposed a Simultaneous Localization and Mapping (SLAM) solution to localize pedestrians through three UWB auxiliary nodes in the case of unknown prior information with the precision to 0.1 m but it needed three nodes to be arranged in advance. Reference [15] proposed a framework of indoor autonomous robot localization using a Sage-Husa fuzzy self-adaptive filter to fuse Inertial Navigation System (INS) and UWB signals. With two UWB nodes deployed, it can perform well with high accuracy and robustness. Reference [16] combines UWB ranging measurements with inertial observations to localize based on Extended Kalman Filter (EKF) with only one auxiliary node but on the assumption of previous knowledge of the position and azimuth of the target node. Fallon et al. proposed a cooperative Autonomous Underwater Vehicle (AUV) navigation method using a single maneuvering surface craft, but it has a direct access to azimuth estimation [17].

A Gaussian white noise is often the presupposition in approaching the nonlinearity of the problem of fusion of IMU and UWB observations. It is well known that most common solutions to nonlinear filtering problems are based on EKF and Unscented Kalman Filter (UKF). However, EKF often suffers large errors and deviations in its estimates of states and variances of non-linear stochastic systems [18], while UKF may miscalculate if the posterior probability density of the system states is non-Gaussian [19]. Compared with EKF and UKF, the main advantage of PF is that they do not place any restrictions on the form of the propagation and measurement models. Owing to the arbitrariness of particle distribution, PF is more suitable for arbitrary non-linear and non-Gaussian stochastic systems, although the computational complexity of PF is relatively large. With the enhancement of computer performance and the development of parallel computing technology, the problem of PF can be overcome [20].

The standard particle filter algorithm uses the resampling method to prevent particle starvation. The specific method is to eliminate particles with smaller weight and copy particles with larger weight. The advantage of this method is simple operation, but after many iterations it will cause lack of particle diversity. If we use ant colony optimization to make particles with smaller weight move to the optimal position, and keep the position of particles with larger weight unchanged, the particles will have better distribution and keep the diversity of particles [21].

In this paper, a cooperative localization method with only one auxiliary node is proposed based on the Adaptive Ant Colony Optimization Particle Filter (AACOPF) [22] and dead reckoning (DR) [23]. Even if the INS suffers power-down and hot reboot, there is no need for initial positioning information to realign itself. Only one auxiliary node is enough to help estimate the real-time position and azimuth of the target node. In the cooperative localization based on ranging information, communication between target nodes and auxiliary nodes as well as that between target nodes themselves is delivered so that the distances between target nodes and auxiliary nodes can be combined with the positions of auxiliary nodes themselves to localize more effectively. The cooperative localization method can establish single-to-multi, multi-to-single, and multi-to-multi network topologies making full use of the nodes in the network, which means, one auxiliary node can locate multiple target nodes whereas one or multiple target nodes may also be located by multiple auxiliary nodes.

Compared with existing approaches, this work has the following innovative aspects:

1. This paper proposes a formulation in which collaborative localization is realized with recourse to only one auxiliary node in motion along with its feasibility proof. Allowing for the multipath effects of UWB and NLOS coupled with occlusion by buildings and pedestrians, an AACOPF algorithm is designed which recognizes and eliminates auxiliary nodes with larger errors in ranging. The algorithm can be applied to Gaussian or non-Gaussian nonlinear models, implying a higher degree of positioning accuracy and robustness.
2. When the topology network containing more than one auxiliary node, the particle filter is able to perform collaborative localization by virtue of adaptive weight adjustment, no extra treatment is needed with the addition of other sensors, thereby permitting a plug and play mechanism, as opposed to traditional schemes bases on the EKF and UKF which, in this situation, necessitate re-linearization or even remodelling.
3. The system has been verified not only by simulation but through real data garnered in real situation and effects including errors in the monitoring of human gait and measurement errors have been taken into account, all of which demonstrate the feasibility and generality of the system.
4. To qualitatively and quantitatively analyse the observability of our cooperative localization system, different from the method in Fallon’s paper, we applied a Piece-Wise Constant System (PWCS) method to analyze the observability of the cooperative navigation system, which can not only qualitatively shows the observability based on the rank of Observability Matrix (OM), but also quantitatively presents the degree of observability based on its eigenvalues.

## 2. Problem Statement

### 2.1. Adaptive Cooperative Localization Problem

For the problem studied in this paper, we define node that does not have accurate coordinates or azimuth due to various reasons such as GPS failure, system malfunction, hot restart, etc. and need to be relocated as target node, and nodes that have access to localization information accuracy as auxiliary nodes.

**Figure 2:**
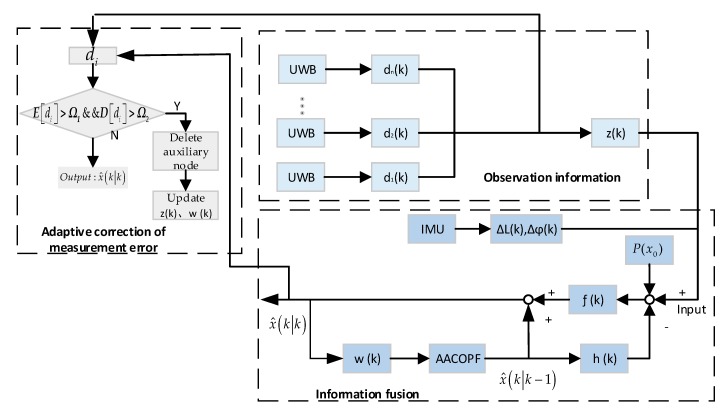
The proposed inertial measurement unit (IMU) and ultra-wideband (UWB) collaborative localization system diagram.

To ensure the observability and convergence of the system, quantitative and qualitative analysis of the observability of our cooperative localization system is necessary. For the dynamical multiple auxiliary nodes topology network, we need to consider the measurement disruption caused by multipath or NLOS effects, particle depletion and dynamical multiple auxiliary nodes data fusion. In the complex collaborative network shown in Figure 1, if a node cannot be self-positioned due to low GPS accuracy or IMU power-down restart, the algorithm of Figure 2 is triggered to detect surrounding auxiliary nodes with UWB information, and combines IMU incremental information from the target node to decide on a co-location algorithm and then achieve coordinated positioning by AACOPF.

There are four presumptions based on which the proposed cooperative navigation algorithm can work properly: (1) Auxiliary node has higher localization accuracy. (2) The target node does not need the initial coordinates or the azimuth, but it is required to have dead reckoning ability. (3) Each node in the network of cooperative localization is capable of ranging with the auxiliary node. (4) Wireless communication is available between nodes to ensure the transmitting of ranging and localization information.

The kinematic model of the cooperative localization system

(1) X(t)=ϕ(t−1)X(t−1)+B(t−1)U(t−1)+ν(t−1)

Let $X(t)={\left[P_{x}(t),P_{y}(t),\varphi (t)\right]}^{T}$ denote the 3D navigation state, comprising the position ${\left[P_{x}(t),P_{y}(t)\right]}^{T}$, azimuth φ(t) of the robot X(t) can be estimated by DR model.

The observation equation of the cooperative localization system

(2) Z(t)=H(t)X(t)+ω(t)

where $Z(t)=\left[d_{i}(t)\right]$ represents all the measurements from IMU and UWB up to the current time *t*, *i* represents the *i*-th sensor. In our positioning localization model, the distance d(t) is between the target node and the auxiliary node.

### 2.2. Kinematic Model

**Figure 3:**
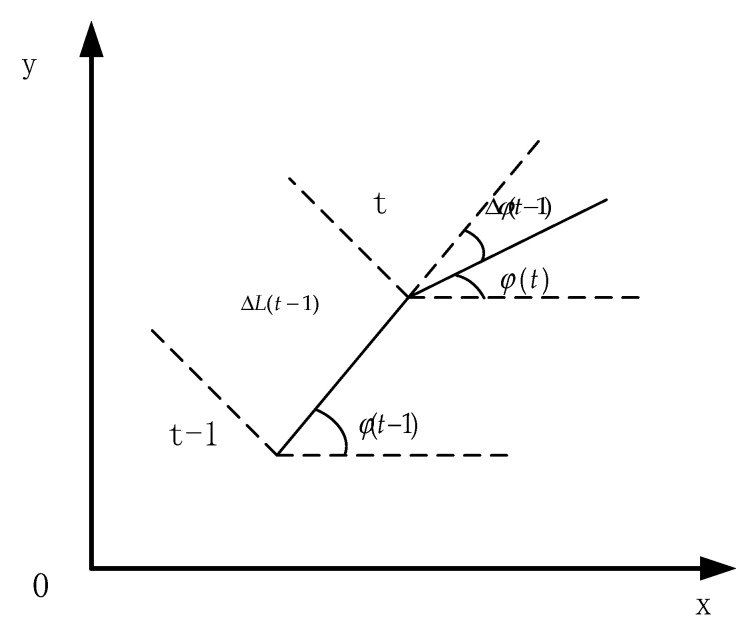
The principle of the dead reckoning (DR) model (the position of the carrier at the next time is estimated using the speed of the velocity, heading, and position of the carrier at the previous time.).

In this paper, we assume that the target node has basic relative dead reckoning capability. The navigation device needs to provide real-time information Δφ(t−1) and displacement increment ΔL(t−1). As shown in Figure 3, this information is used to estimate the current coordinates of the carrier by DR model [24]. At time (*t* − 1), the current position is (x(t−1),y(t−1)). If the next step is to move a distance of ΔL(t−1) with an azimuth angle of φ(t−1) + Δφ(t−1) to the position of time (*t*), the coordinates of time (*t*) can be calculated by Equation (3) [25].

(3) $$\{\begin{array}{c} x(t)=x(t-1)+\Delta L\left(t-1\right)\cos\left[\varphi (t-1)\right]\\ y(t)=y(t-1)+\Delta L\left(t-1\right)\sin\left[\varphi (t-1)\right]\\ \varphi (t)=\varphi (t-1)+\Delta \varphi (t-1) \end{array})$$

The cooperative localization algorithm proposed is insensitive to platforms, no matter they are manned or unmanned. We consider two major types of DR models in this paper. One is based on land vehicle’s odometer and IMU, and the other is based on the shoe-mounted IMU for pedestrian localization.

(1) DR model based on vehicle’s odometer and the IMU

The azimuth angle and displacement incremental information required for co-location is obtained by the Strapdown Inertial Navigation Solution (SINS). Due to the maturity of the development of the SINS based on odometer and IMU, this article does focus much on it. We recommend readers refer to [26].

(2) The DR model for pedestrian localization

From our previous work [27], pedestrian localization is based on the kinematics of the pedestrian, extracting the gait information during pedestrians’ movement, detecting the zero-velocity interval, and triggering the error correction algorithm based on the EKF to achieve the effect of restraining the navigation positioning error. In this paper, velocity information is selected as the observation to establish the current state of the pedestrian, and the EKF is used to suppress the velocity error in the detected zero speed range. Since the observation can only be obtained in the standing period, the EKF only updates the time and measurement information in standing duration (zero speed intervals), and only updates the time in the non-standing period. The state variables of EKF is as follows:

(4) $$X={\left[\begin{array}{cccc} \delta \gamma & \delta \theta & \delta \varphi & \begin{array}{cccc} \delta p_{x} & \delta p_{y} & \delta p_{z} & \begin{array}{ccc} \delta \nu_{x} & \delta \nu_{y} & \delta \nu_{z} \end{array} \end{array} \end{array}\right]}^{Τ}$$

where ${\left[\begin{array}{ccc} \delta \gamma & \delta \theta & \delta \varphi \end{array}\right]}^{Τ}$ are attitude angle errors, $\left[\begin{array}{ccc} \delta p_{x} & \delta p_{y} & \delta p_{z} \end{array}\right]$ are position errors, $\left[\begin{array}{ccc} \delta \nu_{x} & \delta \nu_{y} & \delta \nu_{z} \end{array}\right]$ are speed errors. The linearized system state transition matrix is

(5) $$\Phi_{k,k-1}=\left[\begin{array}{ccc} I_{3\times 3} & 0_{3\times 3} & 0_{3\times 3}\\ 0_{3\times 3} & I_{3\times 3} & \Delta t\times I_{3\times 3}\\ S_{k} & 0_{3\times 3} & I_{3\times 3} \end{array}\right],\;S_{k}=\left[\begin{array}{ccc} 0 & \Delta t\times \alpha_{zk}^{n} & -\Delta t\times \alpha_{yk}^{n}\\ -\Delta t\times \alpha_{zk}^{n} & 0 & \Delta t\times \alpha_{xk}^{n}\\ \Delta t\times \alpha_{yk}^{n} & -\Delta t\times \alpha_{xk}^{n} & 0 \end{array}\right]$$

Taking the actual speed value during standing periods in a pedestrian gait cycle as an observation measurement $Z_{k}=\nu_{k}^{n}-0=\delta \nu_{k}^{n}$, the corresponding observation matrix is $H=\left[\begin{array}{ccc} 0_{3\times 3} & 0_{3\times 3} & I_{3\times 3} \end{array}\right]$, the variance matrix of system process noise and observation noise are defined as $Q=diag\left[\begin{array}{ccc} {\left(0.01\right)}^{2} & {\left(0.01\right)}^{2} & {\left(0.01\right)}^{2}\begin{array}{ccc} \begin{array}{ccc} \begin{array}{cc} 0 & 0 \end{array} & 0 & {\left(0.01\right)}^{2} \end{array} & {\left(0.01\right)}^{2} & {\left(0.01\right)}^{2} \end{array} \end{array}\right]$, $R=diag\left\{\begin{array}{ccc} {\left(0.01\right)}^{2} & {\left(0.01\right)}^{2} & {\left(0.01\right)}^{2} \end{array}\right\}$, respectively.

When the state error estimate of the filter output is brought into the strapdown inertial navigation system for error compensation, then at time *k*, the error-corrected position increment information can be expressed as

(6) $$\Delta L_{k}=p_{k|k-1}-\delta p_{k}-p_{k-1}$$

Attitude error compensation is accomplished by updating the attitude transformation matrix as follows:

(7) $$C_{k}^{n}=\frac{\left(2I_{3\times 3}+\delta \Theta_{k}\right)\cdot C_{k-1}^{n}}{2I_{3\times 3}-\delta \Theta_{k}},\;\;\delta \Theta_{k}=\left[\begin{array}{ccc} 0 & \delta \varphi_{k} & -\delta \theta_{k}\\ -\delta \varphi_{k} & 0 & \delta \gamma_{k}\\ \delta \theta_{k} & -\delta \gamma_{k} & 0 \end{array}\right]$$

The information of the azimuth angle increment after error correction is expressed as follows:

(8) $$\Delta \varphi_{k}=\varphi_{k|k-1}-\delta \varphi_{k}-\varphi_{k-1}$$

### 2.3. Measurement Model Based on UWB

Since the observation model is based on UWB ranging measurements, we analyze the sources of UWB observation errors in order to improve positioning precision. In general, the sources of ranging error are multipath fading $\varepsilon_{m}$ and NLOS propagation $\varepsilon_{nlos}$ [28]. References [29,30] claimed that the propagation error $\varepsilon_{nlos}$ is not relevant to *d*, but rather the penetration coefficient describing how the LOS path is impeded. The distance ranging error $\varepsilon_{d}$ can be expressed explicitly as a function of the Transport-Receive (TX-RX) separation distance and system bandwidth as follows:

(9) $$\varepsilon_{d}=\varepsilon_{m}+\varepsilon_{nlos}$$

At time (*t*), assuming that the position of an auxiliary node in navigation coordinates is $p_{j}(t)={\left[x_{j}(t),y_{j}(t)\right]}^{T}$, where j=1,2⋯n denotes the auxiliary serial number, and *n* is the total number of auxiliary node. Then the measurement distance from the *j*-th auxiliary node with UWB tag to the target node with UWB is written as:

(10) $$d_{1j}(t)=\sqrt{{\left(x_{1}(t)-x_{j}(t)\right)}^{2}+{\left(y_{1}(t)-y_{j}(t)\right)}^{2}}+\varepsilon_{d}$$

(11) $${\hat{d}}_{1,k}^{i}(t)=\sqrt{{\left({\hat{x}}_{1,k}^{i}(t)-x_{j}(t)\right)}^{2}+{\left({\hat{y}}_{1,k}^{i}(t)-y_{j}(t)\right)}^{2}}$$

where the distance $d_{12}\left(t\right)$ between true target node and *j*-th auxiliary node can be measured by ranging sensor, ${\hat{p}}_{1,k}^{i}(t)$ is the position of the *i*-th particle, and the distance ${\hat{d}}_{1,k}^{i}(t)$ between the particle ${\hat{p}}_{1,k}^{i}(t)={\left[{\hat{x}}_{1,k}^{i}(t-1),{\hat{y}}_{1,k}^{i}(t-1)\right]}^{T}$ and the auxiliary node $p_{j}(t)={\left[x_{j}(t),y_{j}(t)\right]}^{T}$ can also be obtained. The particle weight updating equation is established by taking the deviation between ${\hat{p}}_{1,k}^{i}(t)$ and $p_{j}(t)$.

### 2.4. Algorithm Overview 

The cooperative localization method based on AACOPF and dead reckoning is proposed that performs without prior knowledge of the initial location and azimuth of the target node and with only one auxiliary node capable of localization and one ranging sensor measuring the Euclidean distance between a target node and an auxiliary node. The AACOPF algorithm is designed to solve the effects of UWB-related peak offset error and non-Gaussian error caused by UWB multipath and NLOS factor on cooperative localization error in the multiple auxiliary nodes topology network structure. It can realize multi-to-single collaborative localization by adjusting the weight ω(k) update adaptively. It has the function of plug and play, because the new auxiliary node is only an additional source added to the weight update. Thus, if the auxiliary node cannot be used due to signal loss or sensor failure, the system only needs to avoid adding relevant factors, without special procedures to re-model and re-linearize the system model.

## 3. Cooperative Localization Algorithm with an Adaptive Ant Colony Optimization Particle Filter

PF is another non-parametric implementation of Bayesian filtering, whose notion is to represent posterior by a series of random state samples obtained from posteriors. In PF principle, let $\chi_{t}$ be a sample of posteriors distribution (particles), defined as $\chi_{t}:=x_{t}^{\left[1\right]},x_{t}^{\left[2\right]},\cdots ,x_{t}^{\left[m\right]}$, and each particle $x_{t}^{\left[m\right]}(1\leq m\leq M)$ is a possible hypothesis of the state at time *t*. The intuitive sense of PF is to approximate confidence $bel(x_{t}^{m})$ with a series of particles $\chi_{t}$. Ideally, the probability that state $x_{t}$ is included in particles set $\chi_{t}$ is proportional to its posterior $bel(x_{t}^{m})$ of Bayesian filter: $x_{t}^{\left[m\right]}\cdot p(x_{t}|z_{1:t},u_{1:t})$, where $u_{t}$ is the control and $z_{t}$ is the observation [31].

**Figure 4:**
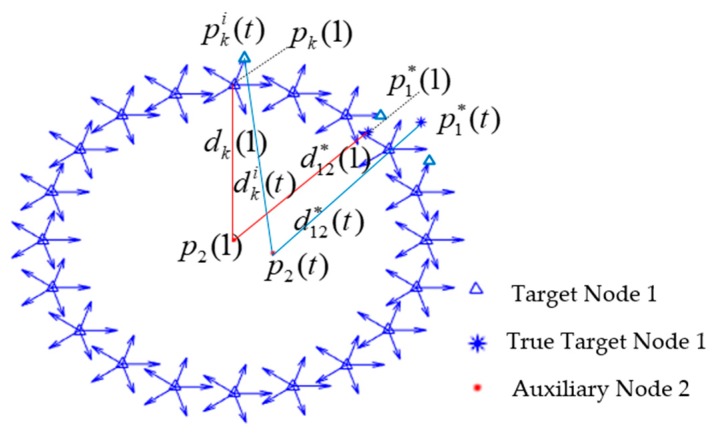
The set of possible locations and initial azimuth of target node 1, and the particles propagation model of target node 1 and auxiliary node 2.

As shown in Figure 4, at time (1), $p_{2}(1)$ represents the position of auxiliary node 2 with normal localization function, $p_{1}^{*}(1)$ represents the true position of target node 1. $d_{12}^{*}(1)$ denotes the measured distance between true target node 1 and auxiliary node 2 obtained by ranging sensor. More precisely, with $p_{2}(1)$ as the center of the circle and $d_{12}^{*}(1)\pm \Delta d$ as the radius. The N×M particles ${\hat{p}}_{1,k}^{i}(1),k=1,2,\ldots N,i=1,2,\ldots M$ of the target node 1 are generated at the time (1) and each particle represents the possible location and initial azimuth of the target node 1. After time (1), N×M particles are propagated by DR model.

Let $d_{12}^{*}(t)$ be the measured true distance between true target node 1 and auxiliary node 2 at (*t*) time. The predicted distance between ${\hat{p}}_{1,k}^{i}(t)$ and auxiliary node 2 is denoted as ${\hat{d}}_{1,k}^{i}(t)$. $\left|\left({\hat{d}}^{i}{}_{1,k}(t)-d_{12}^{*}(t)\right)\right|$ denotes the difference between the predicted distance and the measured distance of each particle, which is used in the calculation of particle weight. The particle with predicted distance that best matches the measured distance gets the highest weight. Through PF, the particles swarm will gradually converge to the true state. The position of the target node 1 is replaced by the position of cooperative localization to realize the trajectory tracking of the target node 1, and the initial azimuth of the target node 1 is derived from the optimal trajectory.

In this paper, ant colony optimization is used to improve the traditional particle filter algorithm. The steps are as follows.

Step 1: Particles initialization

Through the known prior probability density distribution $P(x_{0})$ of the random dynamic system, the initial particles $\left\{x_{0}^{\left(i\right)}\right\},i=1,2,3\cdots N\times M$ at (*t* = 0) are obtained by N×M samples and the corresponding initial weight of each particle is $\omega_{0}^{\left(i\right)}=1/(N\times M)$.

Step 2: Importance sampling

According to $x_{t}^{i}\sim q\left(x_{t}^{i}|x_{t-1}^{i},h\left(t\right)\right)$, i=1,2,3⋯N×M, the particle set $x_{t}^{i}$ at time (*t*) is obtained by the Equation (3).

Step 3: Adaptive cooperative localization

The error in ranging observations are modelled as a Gaussian distribution. In view of the multipath and ULOS effects of UWB and occlusion by pedestrians and buildings along with their consequent impacts on UWB peak values. Adaptive ranging recognition is involved to detect and eliminate auxiliary nodes with unacceptable errors. Updating the weights in the Step 4 leads to higher accuracy and robustness.

Let $\rho_{j}$ denote the residual between the measured distance $d_{1j}$ and the one calculated with estimated position. If the mean value of ranging error is greater than the threshold value $\Omega_{1}$ and the variance is greater than threshold $\Omega_{2}$, that is $E\left[\rho_{j}\right]>\Omega_{1}\&D\left[\rho_{j}\right]>\Omega_{2}$, the node will be eliminated followed by weight updating.

Step 4: Update weights

The predicted value of each particle is obtained by Equation (12), and the weight of each particle $x_{t}^{i}$ is calculated by the following equation according to the current observation $z_{t}$:

(12) $$\omega_{t}^{i}=\omega_{t-1}^{i}\frac{p\left(z_{t}|x_{t}^{i}\right)p\left(x_{t}^{i}|x_{t-1}^{i}\right)}{q\left(x_{t}^{i}|x_{t-1}^{i},z_{t}\right)}$$

In order to get better tracking effect with the ant colony algorithm, it is necessary to eliminate the particles whose estimated values contradict the real value. That is, when $x_{t}^{i}\cdot x<0$, the weight is given $\omega_{t}^{i}=0$; otherwise, the weight remains unchanged.

Normalized particle weights

(13) $$\omega_{t}^{i}=\frac{\omega_{t}^{i}}{\sum_{j=1}^{N}\omega_{t}^{j}}$$

Step 5: Ant colony optimization resampling [32]

The transition probability set of ${\left\{P_{ij}(t)\right\}}_{j=1}^{N\times M}$ from the *i*-th particle to the *j*-th particle at time (*t*) is obtained by the Equation (14). In particle selection, a threshold value λ is set, when the maximum transfer probability $P_{ij}^{best}(t)$ is less than λ, the particle does not change its position. When it is greater than λ, let ${\bar{x}}_{t}^{i}=x_{t}^{j}$, the particle set ${\left\{{\bar{x}}_{t}^{i}\right\}}_{i=1}^{N\times M}$ is updated to maintain particle diversity, and the corresponding weight set of each particle is {ωti}i=1N×M=1N×M. The value of λ can be obtained by experiments or experience.

(14) $$P_{ij}(t)=\frac{{\hat{\omega }}_{ij}^{\alpha }(t)\eta_{ij}^{\beta }(t)}{\sum {\hat{\omega }}_{ij}^{\alpha }(t)\eta_{ij}^{\beta }(t)}$$

Define the particle transition probability $P_{ij}(t)$ to represent the probability that particle *i* will shift to *j* at the time (*t*) and ${\hat{\omega }}_{ij}^{\alpha }(t)$ denote the difference between the weights of particle *i* and *j* at the time (*t*). The greater the difference between weights, the greater the probability that particle *i* will move to particle *j*. $\eta_{ij}^{}(t)$ is the moving distance of the particles. The shorter the distance the particles move, the greater the probability. *α* and *β* respectively reflect the weight information and position information of the particles during the transfer process.

Step 6: Output state estimation

According to the steps above, the current state of target node 1 can be estimated by Equation (15), and let *t* = *t* + 1, return to Step 2.

(15) $${\hat{x}}_{t}=\sum_{i=1}^{N\times M}\omega_{t}^{i}{\bar{x}}_{t}^{i}$$

At time (*t*), the particle with the largest weight among all particles is selected and its corresponding particles index *L* is calculated by the following equation:

(16) L=r(modm)

where, in Equation (16), m represents each initial particle have *m* directions, and *r* is the index of the largest weight particle.

## 4. Observability Analysis of Cooperative Localization System with One Auxiliary Node

For discrete time-varying linear systems, Goshen-Meskin et al. proposed a Piece-Wise Constant System (PWCS) observability analysis theory [33]. According to the motion characteristics of the system, the system can be divided into time segments and the observability matrix of each time segment can be calculated, so that the total observability matrix of the system can be obtained. The following introduces an observability analysis method based on singular value decomposition of the observability matrix of the system.

Without considering the process noise and the observation noise, the linearized system model is:

(17) $$\begin{array}{l} \Delta X(t)=\Phi \Delta X(t-1)+B\Delta u(t-1)\\ \Delta Z(t)=H\Delta X(t) \end{array}$$

where,

$$\Phi =\left[\begin{array}{ccc} 1 & 0 & -\Delta L\left(t-1\right)\sin\left[\varphi (t-1)\right]\\ 0 & 1 & \Delta L\left(t-1\right)\cos\left[\varphi (t-1)\right]\\ 0 & 0 & 1 \end{array}\right],B=\left[\begin{array}{cc} \cos\varphi (t-1) & 0\\ \sin\varphi (t-1) & 0\\ 0 & 1 \end{array}\right],H=\left[\begin{array}{ccc} \frac{x(t)-x_{2}(t)}{d(t)} & \frac{y(t)-y_{2}(t)}{d(t)} & 0 \end{array}\right]$$

For a set of observations H(0),H(1),H(2),⋯,H(k), based on the observability analysis theory of PWCS, the observability matrix of dynamic system is:

(18) $$R_{k}=\left[\begin{array}{c} H(0)\\ H(1)\Phi (0)\\ H(2)\Phi (1)\Phi (0)\\ \vdots \\ H(K)\prod_{i=0}^{k-1}\Phi (i) \end{array}\right]$$

where ϕ is the state transition matrix of the system, and H is the observation matrix.

The singular value decomposition of $R_{k}$ is expressed as:

(19) $$R_{k}=U{\sum V}^{T}$$

where $U=\left[u_{1},u_{2},\cdots ,u_{k}\right]$ is an k×k dimensional orthogonal matrix; $V=\left[v_{1},v_{2},v_{3}\right]$ is a 3×3 dimensional orthogonal matrix; $\sum =\left[S_{3\times 3},0_{(k-3)\times 3}\right]$ is a k×3 dimensional matrix; $S=diag\left(\sigma_{1},\sigma_{2},\sigma_{3}\right)$ is a diagonal matrix composed of the singular value of $R_{k}$ and 0.$\sigma_{1}\geq \sigma_{2}\geq \cdots \geq \sigma_{r}>\sigma_{r+1}=\cdots =\sigma_{n}=0,n=3$. The singular value $\sigma_{i}$ of $R_{k}$ is greater than 0. The magnitude of the singular value can effectively reflect the observability of the state of the system. The larger the singular value, the better the observability of the corresponding state.

**Figure 5:**
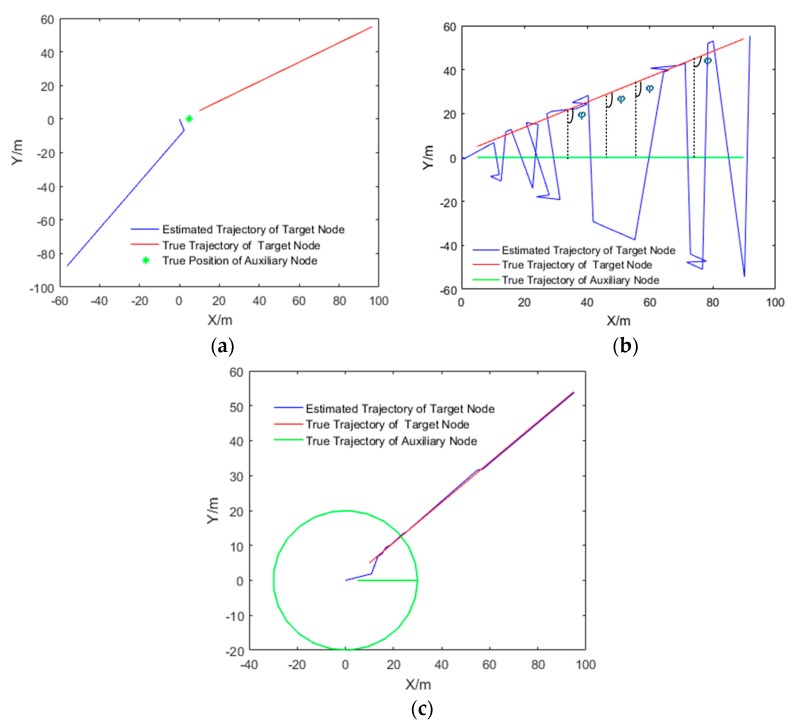
System observability analysis. (**a**) The auxiliary node fixed (red dot), (**b**) the range of target node to the auxiliary node (red line) located at the same relative direction, (**c**) the auxiliary node (red line) does not move with respect to the target node.

Gadre and Stilwell [34] proposed that the system is locally unobservable when the auxiliary node is fixed or the range of target node to the auxiliary node located at the same relative direction. According to the observability analysis theory of PWCS, the observability of the system is determined by singular value σ which is calculated by $R_{k}$. Suppose the target node (blue line) makes a linear motion with a slope of 0.5, the estimated trajectory of target node is shown in Figure 5.

As shown in Table 1, when the auxiliary node is fixed or the range of target node to the auxiliary node located at the same relative direction, there is a singular value of 0, which denotes the system is not observable. When the auxiliary node does not move with respect to the target node, the system is observable for the full rank of observable matrix $R_{k}$. Therefore, to make the system observable, in the process of cooperative localization, the PWCS observability analysis method can be used to optimize the appropriate auxiliary node trajectory.

**Figure 6:**
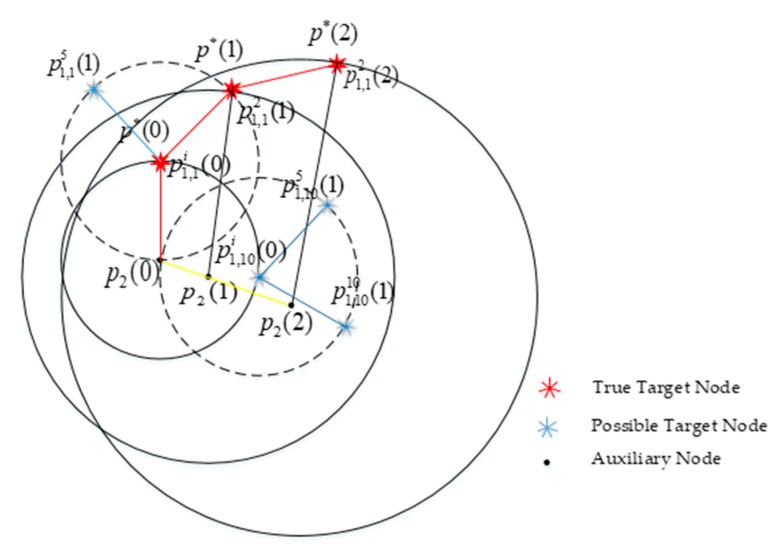
Particle’s propagation model of target node 1 with a moving auxiliary node.

According to PF algorithm and DR model, if auxiliary node 2 is moving, the particles propagation of the AACOPF algorithm is shown in Figure 6. $p_{1,k}^{i}\left(t\right)=\left[x_{1,k}^{i}\left(t\right),y_{1,k}^{i}\left(t\right)\right]$ (k=1,2,…,N,i=1,2,3,…,m) are the possible positions of target node 1. $p_{}^{*}\left(t\right)=\left[x_{}^{*}\left(t\right),y_{}^{*}\left(t\right)\right]$ are the true position of target node 1, and $p_{2}\left(t\right)=\left[x_{2}\left(t\right),y_{2}\left(t\right)\right]$ denotes the position of moving auxiliary node 2. At time (0), N particles are generated, and each particle is given m directions, after particles propagate by DR model, we can get N×M particles at time (1). The red line denotes the true trajectory of target node 1, the blue line is the estimated trajectory, and the yellow line denoted the true trajectory of auxiliary node 2. According to the proof of cooperative positioning algorithm, only when node 2 is in motion, the error converges, thus, the coordinates of the target node 1 can be estimated.

The cooperative localization algorithm is shown in Algorithm 1. By making use of the known information, such as the distance $d_{12}^{}(t)$ between target node and auxiliary node, weight information *α*, position information *β*, threshold value λ, displacement increment ΔL(t), and azimuth increment Δφ(t) of target node, we can get the localization information ${\tilde{p}}_{1}^{}(t)$ and initial azimuth $\varphi_{1,k}^{i}\left(0\right)$ of the target node. In Table 1, ${\hat{p}}_{1,k}^{i}(t)=\left[x_{1,k}^{i}\left(t\right),y_{1,k}^{i}\left(t\right)\right]$ denotes the predicted state of particle *k* with initial azimuth *i* at time (*t*).
**Algorithm 1** Cooperative Localization Algorithm of Single Auxiliary Node1: **Input:** Number of initial particles *N*, Number of initial azimuth *M*, Euclidean distance $d_{12}^{}(t)$, Displacement increment ΔL(t), Azimuth increment Δφ(t) 2: **Output:** Estimation trajectory ${\tilde{p}}_{1}^{}(t)$, azimuth $\varphi_{k}^{i}\left(t\right)$
3: **Generated**
N×M
**initial particles**
$p_{N}^{m}\left(0\right)$
4: **While** localization **do** 5:   **for** 0 < *k* < *N*
**do** 6:     **for** 0 < *i* < *M*
**do** 7:        $x_{k}^{i}\left(t\right)\leftarrow x_{k}^{i}\left(t-1\right)+\Delta L\left(t\right)\cos\left[\varphi_{k}^{}\left(t-1\right)+\Delta \varphi \left(t\right)\right]$ 8:        $y_{k}^{i}\left(t\right)\leftarrow y_{k}^{i}\left(t-1\right)+\Delta L\left(t\right)\sin\left[\varphi_{k}^{}\left(t-1\right)+\Delta \varphi \left(t\right)\right]$ 9:     **end** 10:   **end** 11: ${\hat{d}}_{k}^{i}(t)\leftarrow \sum_{j=1}^{n}\left\|{\hat{p}}_{k}^{i}(t)-p_{j}(t)\right\|$ 12:   **Particles weight**
$w_{k}^{i}\leftarrow sort(\frac{\exp{\left(\sum_{j=1}^{n}\left({\hat{d}}_{k}^{i}(t)-d_{1j}^{}(t\right)\right)}^{2})}{\sum \exp{\left(-{\hat{d}}_{k}^{i}(t)-d_{1j}^{}(t)\right)}^{2}}))$ 13:   **Moving distance**
$\eta_{k}^{ij}(t)\leftarrow \frac{1}{norm(p_{k}^{i}-p_{k}^{j})}$ 14:   **Transition probability**
$p_{ij}=\frac{w_{ij}^{\alpha }(t)\eta_{ij}^{\beta }(t)}{\sum w_{ij}^{\alpha }(t)\eta_{ij}^{\beta }(t)}$ 15:   **If**
$p_{ij}^{\max}>\lambda$      ${\bar{p}}_{k}^{i}=p_{k}^{j}$ 16:   **end** 17:   **Estimation trajectory**
${\tilde{p}}_{1}^{}(t)\leftarrow mean(\sum_{i=1}^{3}w_{k}^{i}{\hat{p}}_{1}^{i}(t))$ 18:   **Localization** error $e_{1}\left(t\right)\leftarrow \left\|{\tilde{p}}_{1}\left(t\right)-p_{1}^{*}\left(t\right)\right\|$ 19:   **Azimuth**
$\varphi_{k}^{i}\left(t\right)\leftarrow \left[\varphi_{k}^{i}\left(t-1\right)+\Delta \varphi \left(t\right)\right]$ 20: **end**

## 5. Experiment and Results

### 5.1. Car-to-Trolley Cooperative Localization

#### 5.1.1. Experimental Sets 

**Figure 7:**
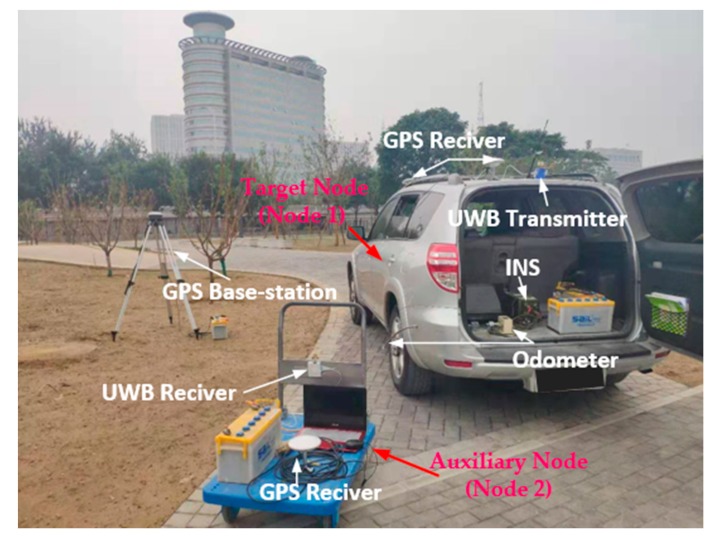
Car-to-trolley cooperative localization system.

In this section, firstly, we introduce the platform of the experiment and then analyze the feasibility of the algorithm. The platform consists of a car equipped with an odometer, IMU, GPS, and UWB transmitter, and a trolley with UWB receiver and GPS. The objective of the mission is to efficiently verify the dual-platform localization algorithm based on DR and AACOPF, then, analyze the tracking error of the car. The experimental setup and picture is shown in Figure 7.

The experimental sets are as following:

1. The distance between the car and the trolley is measured by UWB ranging module (DW1000FOLLOWER). The measurement accuracy of DW1000 chip is about 10 cm, and the bidirectional ranging accuracy is 20–30 cm.
2. The car equipped with IMU, odometer, GPS, and UWB ranging module functions as the target node (node 1). The trolley functions as auxiliary node (node 2), which is equipped with GPS that can provide accurate position information as well as UWB ranging module. The positioning accuracy of GPS is 5 cm, and the Gyro bias less than 0.05°/h.
3. The displacement increment ΔL(t) and azimuth increment Δφ(t) of the car is obtained by odometer and INS. On the car, the double antenna GPS is not used in the localization process but regarded as reference criterion to verify the accuracy of cooperative localization algorithm.
4. The position of the trolley is measured by GPS.
5. The GPS localization information of the car is assumed to be the benchmark, and the localization information calculated by cooperative localization is the estimated information.
6. The experimental site covers an area of approximately 100 × 100 m^2^. The car moves in a closed loop around the site.

#### 5.1.2. Results 

(1) Tracking Verification When the Trolley is in Static and Moving State

**Figure 8:**
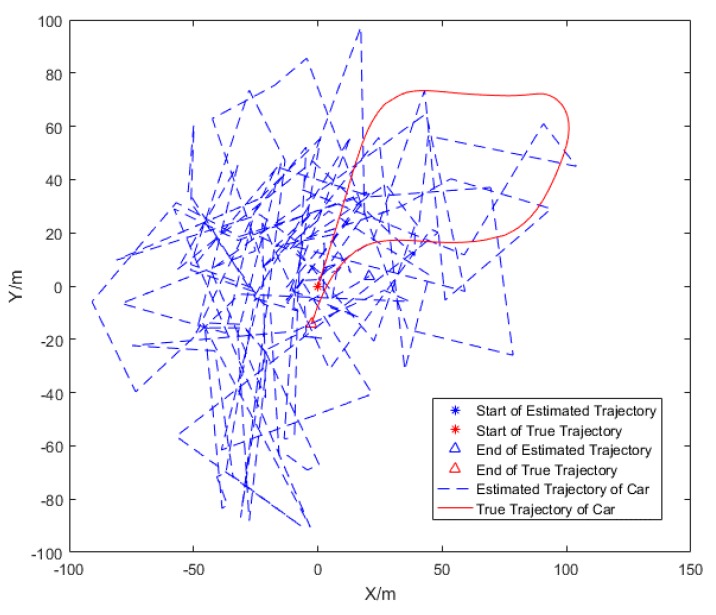
The trajectory tracking of car (target node 1) with trolley stationary (auxiliary node 1).

**Figure 9:**
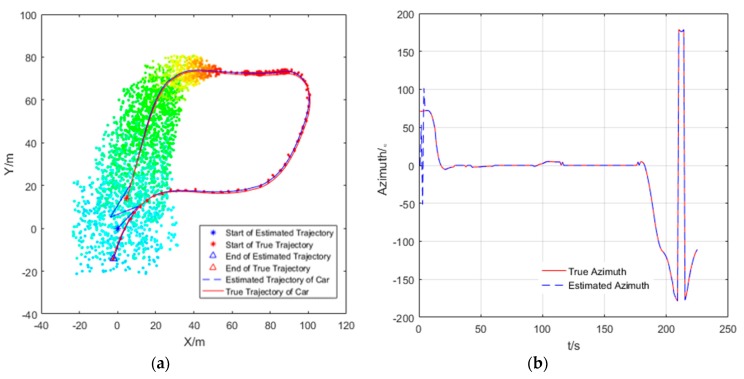
The trajectory tracking (**a**) and azimuth (**b**) estimation of car (target node 1) with trolley moving (auxiliary node 1).

The estimated trajectory and the actual trajectory of the car is drawn in Figure 8 and Figure 9. The trolley remains stationary in Figure 8. In Figure 9, the motion of the trolley satisfies the observable motion of the system. The feasibility of the algorithm is verified by comparing the estimated trajectory with the actual trajectory.

**Figure 10:**
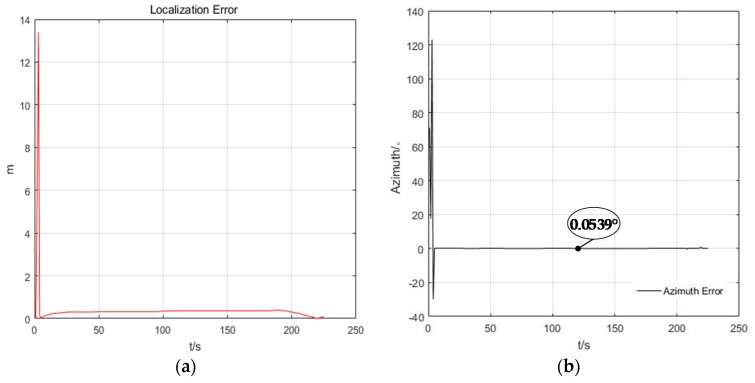
The positioning (**a**) and azimuth (**b**) estimation error of the car (target node 1) for one of the running program, when *N* = 400, *M* = 400.

In the experiment, the true initial azimuth of car is φ=71.0548°. In Figure 8, it can be seen that when the trolley is stationary, the estimation trajectory of car cannot track the true trajectory, and the estimation of initial azimuth $\varphi_{1}^{i}=11.9977{}^{\circ}$ is very different from the true initial azimuth φ=71.0548°, the cooperative localization algorithm could neither track the trajectory of the car, nor can it estimate the azimuth when the trolley is stationary. However, in Figure 9, when the trolley is moving, the estimated trajectory and azimuth of the car is convergent, and the convergence process of particles at different times is represented by different colors. In Figure 10, it can be seen that the cooperative localization algorithm can track the trajectory of the car with high accuracy of up to 0.3 m. the initial azimuth $\varphi_{1}^{i}=71.0009{}^{\circ}$ calculated by the algorithm and the estimated the azimuth error converges and the accuracy of azimuth estimation reaches 0.0539°.

(2) Select the Appropriate Localization Parameters on the Estimation of States

It can be seen from Table 2 that the accuracy of the positioning error and azimuth error keep stable after *N* = 400 and *M* = 400. In general, the larger the value of *M* and *N* are, the smaller the positioning error and the azimuth error are. However, there are some special cases, such as when *N* = 100 and *M* = 100, the system has high accuracy, which are caused by the formation of N×M particles (random generation in this paper) and UWB ranging error. Ultimately, considering the positioning accuracy and the azimuth estimation accuracy, *N* = 400 and *M* = 400 can be selected as the relatively appropriate trajectory parameters in the 3D circumstance.

(3) Comparison of Positioning Accuracy of EKF, PF, and AACOPF with Gaussian Ranging Noise

**Figure 11:**
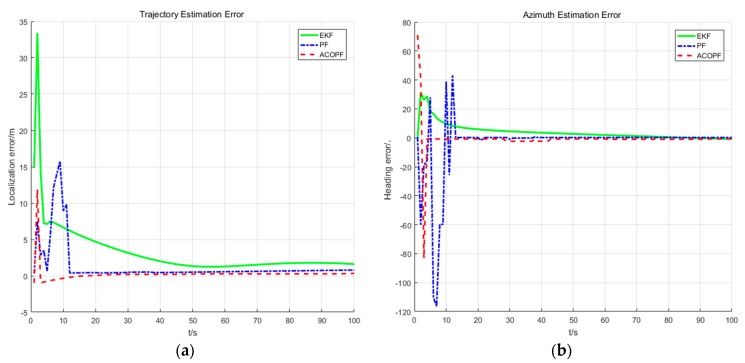
The positioning (**a**) and azimuth (**b**) estimation error of the car (target node 1) for one of the running programs, when *N* = 400, *M* = 400.

The localization errors and azimuth estimation errors of the pedestrian in Figure 11 show that the convergence and localization accuracy of cooperative positioning are EKF < PF < AACOPF. The reason for this phenomenon is that our co-localization model is a nonlinear system. The EKF principle is to linearize the nonlinear system, which often suffers large errors and deviations in its estimates of states and variances. Owing to the arbitrariness of particle distribution, the PF is more suitable for arbitrary non-linear systems. The standard PF algorithm uses the resampling method to prevent particle starvation. One of the advantages of this method is simple operation, but after many iterations it will suffer from lack of particle diversity. Just as we can see from the azimuth estimation error in Figure 11, although PF has fast convergence rate and high positioning accuracy, its curve experiences some protruding outliers. Therefore, we use AACOPF to make the particles have better distribution and keep the diversity of particles.

### 5.2. Car-to-Pedestrian Cooperative Localization

#### 5.2.1. Experimental Sets

**Figure 12:**
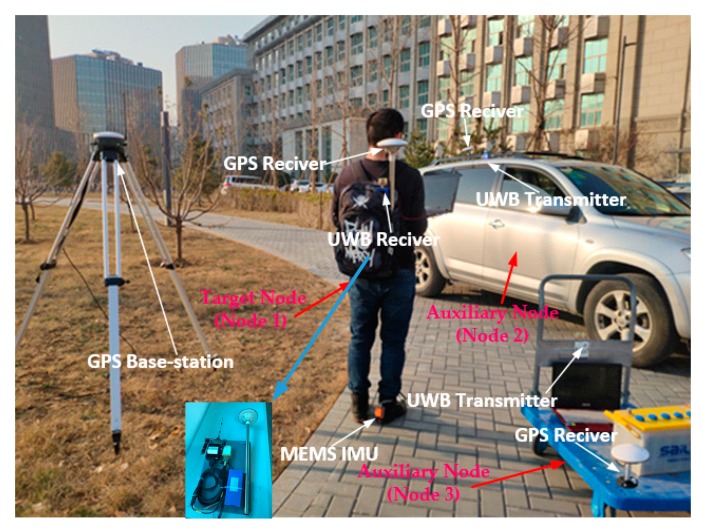
Experimental equipment of MTi-G710.

As Figure 12 has shown, the MTi-G710 inertial measurement unit of Xsens of the Netherlands is selected as the experimental equipment. The IMU is 57 mm × 42 mm × 24 mm in size and only 21 g in weight, so it has the characteristics of small and light, which is very suitable for foot installation and will not affect the normal action of pedestrians. MTi-G710 mainly integrates MEMS gyroscope, MEMS accelerometer and a three-axis magnetometer. The gyroscope bias of less than 5°/h and acceleration bias is 12 ug. In the field of 30 m × 15 m, pedestrians wear the shoes of a fixed IMU and hold the laptop connected with IMU and UWB. The inertial data measured by IMU is processed by the strapdown inertial navigation solution based on zero-velocity updating. The GPS on backpack is regarded as reference criterion to verify the accuracy of cooperative localization algorithm. The car and trolley function as auxiliary nodes (node 2 and node 3), which are equipped with GPS that can provide accurate position information as well as UWB ranging module. In the course of the experiment, the auxiliary node 2 is added with the peak shift error, the auxiliary node 3 is added with non-Gaussian noise or the noise that exceeds the measurement error threshold.

#### 5.2.2. Results 

Comparison of positioning accuracy of PF and AACOPF with non-Gaussian ranging noise.

**Figure 13:**
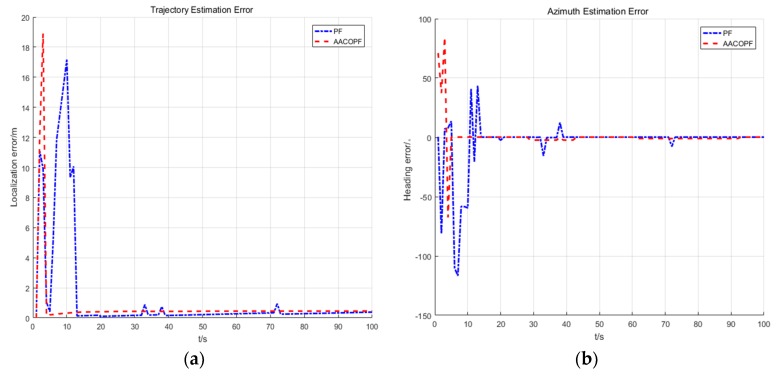
The trajectory estimation errors (**a**) and azimuth estimation errors (**b**) of the pedestrian (target node 1) for one of the running programs, when *N* = 400, *M* = 400.

When auxiliary node 2 is added with the peak shift error, auxiliary node 3 is added with non-Gaussian noise or the noise that exceeds the measurement error threshold, the localization errors and azimuth estimation errors of the pedestrian in Figure 13 show that AACOPF convergences faster than PF, and the localization accuracy is higher than PF. Due to non-Gaussian ranging noise and particle starvation of PF, we can see from the curve of azimuth estimation error and trajectory estimation errors in Figure 12 that PF experiences some protruding outliers, while the AACOPF not. Therefore, we use AACOPF to make the particles have better distribution and keep the diversity of particles, through AACOPF the accuracy of localization and azimuth of this system is 0.66372 m and 0.22658°.

## 6. Conclusions

In this paper, a multi-platform cooperative localization method based on IMU and UWB is proposed. Firstly, the particle propagation model is constructed based on a DR model. Then the weight updating algorithm is designed using ranging information between target node and auxiliary node measured by UWB as observations. The position and azimuth of the target node are estimated by AACOPF. The convergence property of the algorithm is proved and analyzed to verify the feasibility and effectiveness of the single to single cooperative localization algorithm. This method contributes to solving the problem of localization in harsh and complex environments. Only one moving auxiliary node is needed to estimate the position and initial azimuth of the target node, with no prior information of position and azimuth of the target node. When there are multiple auxiliary nodes (new sensors are added), the particle filter algorithm can perform rapid cooperative positioning without remodelling or re-linearization of the system, only by self-adaptive adjustment of particle weights. It has the capacity for plug and play operation since a new sensor is only added as a guide of weight updating. Similarly, if the sensor cannot be used due to signal loss or sensor failure, the system only needs to avoid adding relevant factors, without special procedures.

## Figures and Tables

**Table 1:** The singular value of the system state under three kinds of motion states.

	Auxiliary Node Fixed	Relative Movement	Non-Relative Movement
$\sigma_{1}$	1.2884	103.0097	124.9266
$\sigma_{2}$	0.0016	1	4.0294
$\sigma_{3}$	0	0	0.8866

**Table 2:** Effects of the number of initial positions and azimuth on the positioning error and azimuth error.

M	N	Initial Azimuth Error/°	Localization Error/m
50	50	6.9477	4.5267
100	100	0.1134	0.2583
200	200	1.3426	1.2635
400	400	0.0539	0.0687
600	600	0.0684	0.0681
800	800	0.0539	0.0187
1000	1000	0.0539	0.0245
1500	1500	0.0539	0.0197
